# Sleep Apnea and Cardiovascular Diseases

**DOI:** 10.1155/2014/690273

**Published:** 2014-02-05

**Authors:** Rodolfo Álvarez-Sala, Francisco García-Río, Félix Del Campo, Carlos Zamarrón, Nikolaus C. Netzer

**Affiliations:** ^1^Department of Pneumology, La Paz University Hospital, Madrid, Spain; ^2^Instituto de Investigación Sanitaria del Hospital Universitario La Paz (IdiPAZ), Autónoma de Madrid University, Madrid, Spain; ^3^Department of Pneumology, Río Hortega Hospital, Valladolid University, Valladolid, Spain; ^4^Department of Pneumology, Santiago de Compostela Hospital, Santiago de Compostela Universidad, A Coruña, Spain; ^5^Hermann Buhl Institute for Hypoxia and Sleep Medicine Research, Research Program of the Paracelsus Medical University and Clinic Ghersburg for Geriatric Rehabilitation, Bad Aibling, Germany

Obstructive sleep apnea (OSA) is a common respiratory disease characterized by abnormal collapse of the pharyngeal airway during sleep, causing repetitive arousals and falls in the oxygen saturation [1]. This disorder, when not correctly treated, has been associated with higher fatal and nonfatal cardiovascular events [2]. Also, OSA has been related to many cardiovascular diseases (CVD) such as heart failure [3], arrhythmias [4], pulmonary hypertension [5], and coronary artery disease (CAD) [6]. Moreover, in recent years OSA has emerged as a major public health problem due to its profound impact on patients' health. By this reason, we have proposed this special issue about the relationship between sleep disordered breathing (SDB) and CVD. We have suggested different topics such as potential mechanisms linking OSA to CVD, acute cardiovascular effects of OSA, impact of OSA on natural history of CVD, cardiovascular effects of continuous positive airway pressure (CPAP) treatment, and therapeutic cardiovascular efficacy of emerging treatment for OSA and central sleep apnea (CSA).

Several mechanisms are involved in the association between OSA and CVD. In fact, enhanced sympathetic activity, oxidative stress, systemic inflammation, and endothelial dysfunction contribute to this association and promote atherogenesis. Moreover, cyclic decreases in blood oxygen saturation during recurrent episodes of apnea produce chronic intermittent hypoxia and contribute to the development of atherosclerosis. Some hypoxia-induced transcription factors as hypoxia-inducible factor-1 and nuclear factor-K*β* are activated during intermittent hypoxia and contribute to the generation of inflammation and expression of proinflammatory cytokines such as tumor necrosis factor-*α* and interleukin-8.

It seems that these mechanisms could have an important impact in the occurrence of ischemic cardiac events. Hence, CAD in OSA patients probably precludes the activation of multiple mechanisms, such as atherosclerosis, hypertension, and endothelial dysfunction. Likewise, OSA may influence outcomes in patients with acute or chronic CAD, but this effect remains controversial as results obtained from studies have yielded discrepant data. In this way, Yumino et al. [7] observed a higher incident of cardiac death, reinfarction, or new revascularization in OSA patients with CAD and compared to subjects without OSA, after a mean follow-up period of 7 months. Nevertheless, Hagenah et al. [8] found that OSA did not increase the risk of mortality and cardiovascular complications in 50 patients with stable CAD after 10 years of follow-up period. Moreover, concerning treatment of OSA patients with CAD, CPAP may have beneficial effects in recurrence of ischemic cardiac disease and necessity of new revascularization procedures but higher-evidence studies are necessary to investigate the effect of CPAP in these subjects.

Regarding heart failure, a high prevalence of OSA and CSA in this disorder has been observed. Also, different therapeutic strategies such as oxygen therapy, CPAP, bilevel positive airway pressure (BIPAP), and adaptive servoventilation (ASV) have been proposed to treat OSA in heart failure patients. In this way, in OSA heart failure subjects, CPAP may improve survival, left ventricular ejection fraction, and quality of life [9–11]. Nevertheless, in heart failure CSA patients, CPAP probably is not effective as ASV to abolish the apnea-hypopnea index to <15 events·h^−1^ [12]. So far, it is evident that SDB treatment has showed consistent improvements in heart function, quality of life, and respiratory events in patients with heart failure. However, it is necessary to evaluate the impact of these respiratory treatments in randomized-controlled, long-term longitudinal studies.

Finally, respecting the association between atrial arrhythmia and OSA, it has been described that cardiac remodeling, sympathetic activity, and systemic inflammation may lead to the development of arrhythmias in these patients. Moreover, hypoxemia and hypercapnia have an important role in the pathogenesis of this association. However, it is necessary to investigate the effect of CPAP treatment in atrial arrhythmias of OSA patients.

In conclusion, sleep apnea is a high prevalence disease that has an important relationship with cardiovascular disease. In this special issue, we have tried to make an extensive review about mechanisms involved in the association between ischemic cardiac disease, heart failure, and arrhythmias with OSA. Nevertheless, we know that despite multiple reports about OSA and cardiovascular disease, it is yet necessary to perform higher grade evidence studies to explore mechanisms involved in this association and the impact of CPAP treatment in these subjects.

*Rodolfo Álvarez-Sala* *Rodolfo Álvarez-Sala* *Francisco García-Río* *Francisco García-Río* *Félix del Campo* *Félix del Campo* *Carlos Zamarrón* *Carlos Zamarrón* *Nikolaus Netzer* *Nikolaus Netzer*
